# Supplementary material for: Nocturnal Hypoxemia and CT Determined Pulmonary Artery Enlargement in Smokers
Source: J Clin Med. 2021 Jan 30;10(3):489. doi: 10.3390/jcm10030489 (PMC7866508; doi:10.3390/jcm10030489)
Supplement: Supplementary file 1 [file jcm-10-00489-s001.pdf]

Journal of
Clinical Medicine

Table S1. Details of inclusion/exclusion criteria

Inclusion criteria	SAIL Study	CHAIN Study
Tobacco consumption	>30 packs/year	>10 packs/year
Age	55–75 years	>35 years
Informed consent	Yes	Yes
Diagnosis of COPD	Not a criterion	Postbronchodilator $FEV_1/FVC < 0.7$ after 400 μg of inhaled salbutamol
**Exclusion criteria**	None	Bronquiectasis not associated with COPD
Exclusion criteria for this analysis		Previous diagnosis of:<br>• Obesity-hypoventilation syndrome<br>• Neuromuscular disorder<br>• Heart failure<br>• Thoracic aorta aneurism<br>• Interstitial lung disease<br>• Resting Hypoxaemia

Chest CT scan

Measurements were made from axial CT images with the use of inspiratory acquisitions. The diameter of the main pulmonary artery (PAD) at the level of its bifurcation and the diameter of the ascending aorta (Ao) in its maximum dimension was measured using the same images (Figure E1).

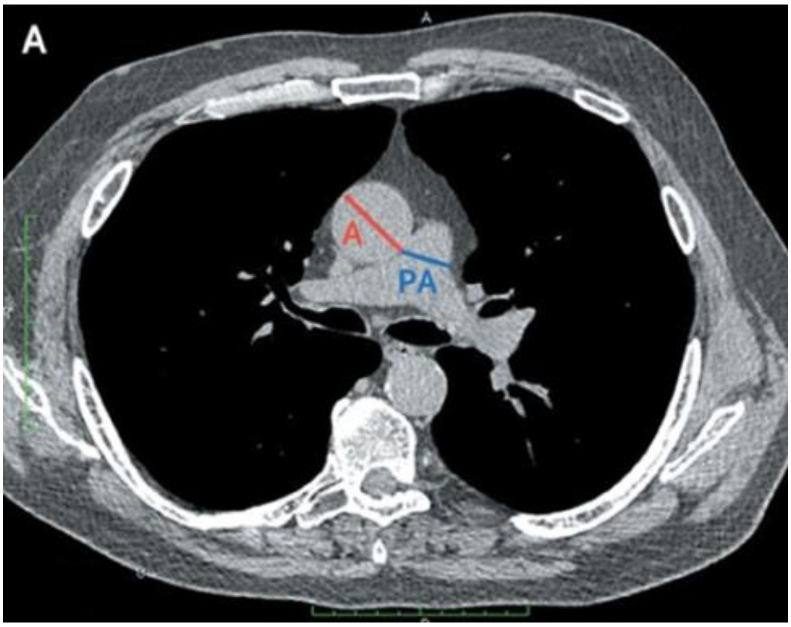

Figure S1. Measurement of the diameters of the main pulmonary artery (PAD) and aorta (AD). Figure shows trans axial chest computed tomographic at the level of the left and right main pulmonary arteries. The ratio of PAD and AD were measured at the level of the bifurcation of the main pulmonary artery.

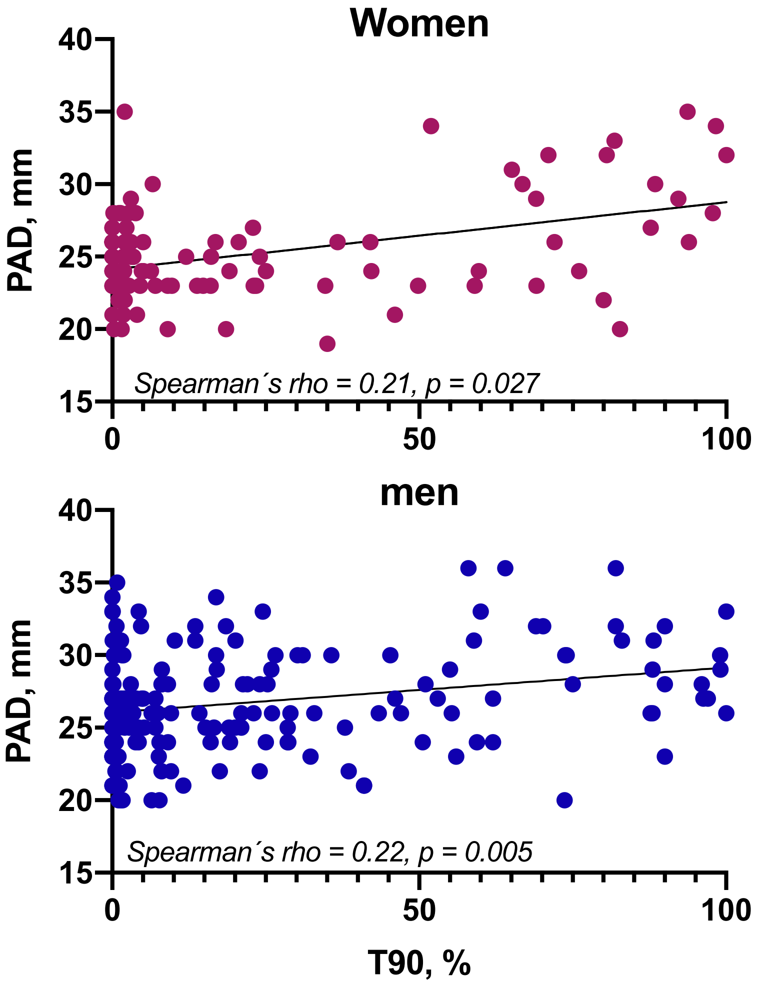

Figure S2. Relationship of percentage of time spend with $SpO_2 < 90\%$ during sleep recording (T90) with pulmonary artery diameter (PAD) in women (*upper panel*) and men (*lower panel*). Positive correlation was higher in men than women (Spearman's rank correlation coefficient).

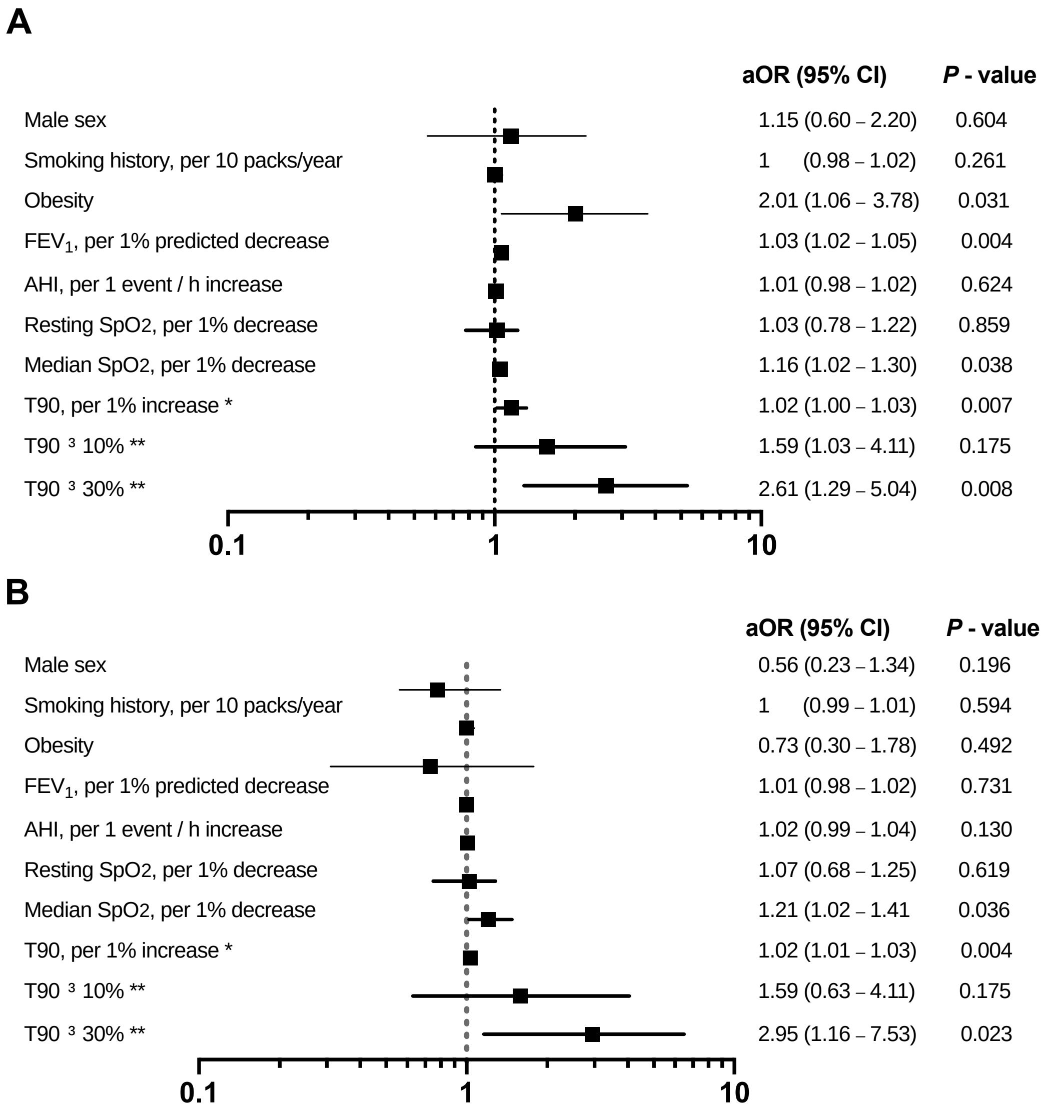

Figure S3. Forest plots of risk factors associated with pulmonary artery enlargement in participants without obstructive sleep apnoea as defined by the main pulmonary artery diameters (**A**) and by the PA:Aa ratio (**B**). Variables in the multivariate regression model included: age, gender, smoking status (current vs. ex-smoker), smoking history, obesity, FEV_1 % predicted, apnoea–hypopnoea index, resting O_2 saturation, T90 and centre site. Abbreviations: aOR = adjusted odds ratio; CI = confidence interval, FEV1 = forced expiratory volume in one second, AHI = apnoea–hypopnoea index; T90 = percentage of sleep recording time with oxygen saturation <90%. * OR estimates in the fully adjusted model instead of median O_2 saturation. ** OR estimates in the fully adjusted model instead of median O_2 saturation and T90.

Table S2 Characteristics of Participants in the SAIL and CHAIN cohorts *

Variable	SAIL Cohort (*n* = 210)	CHAIN Cohort (*n* = 74)	*p*-Value *
**Male**, *n*(%)	126 (60)	52 (70)	0.983
**Age**, years-mean (SD)	66 (6)	63 (10)	0.004
**Smoking history**			
Packs/year	45 (38–60)	40 (20–50)	0.001
Current smokers, *n*(%)	113 (54)	29 (39)	0.031
**Body mass index**, kg/m^2	28 (25–31)	27 (24–30)	0.563
**Prevalent comorbidities**, *n*(%)			
Hypertension	82 (39)	29 (39)	0.681
Diabetes	27 (13)	13 (18)	0.317
Hyperlipidemia	69 (33)	20 (27)	0.353
Coronary heart disease	17 (8)	11 (15)	0.093
Atrial fibrillation	13 (6)	6 (8)	0.570

**COPD**, *n* (%)	109 (52)	49 (66)	0.041
**OSA**, *n* (%)	127 (60)	38 (51)	0.174

Definition of abbreviations: SAIL = Sleep Apnoea in Lung Cancer Screening Study; CHAIN = COPD History Assessment in Spain; Post-BD FEV_1 = post-bronchodilator forced expiratory volume in the first second; COPD = chronic obstructive pulmonary disease; OSA = obstructive sleep apnoea as defined by an apnoea–hypopnoea index ≥ events·h $^{-1}$. Data are shown as median (interquartile range), mean (SD) or number (%). * *p*-value differences were assessed using t tests or Mann–Whitney test for continuous variables and Pearson's Chi tests for categorical variables.

Table S3. Characteristics of participants grouped by the presence of pulmonary artery enlargement as assessed by PA:Ao ratio *

Variable	All Subjects (*n* = 284)	PA:Ao Ratio ≤ 0.9 (*n* = 252)	PA:Ao Ratio > 0.9 (*n* = 32)	*p*-Value *
**Male**, *n*(%)	178 (63)	161 (64)	17 (50)	0.236
**Age**, years -mean (SD)	65 (7)	65 (7)	65 (6)	0.861
**Smoking history**				
Packs/year	42 (35–60)	42 (35–60)	43 (33–60)	0.623
Current smokers, *n*(%)	142 (50)	128 (51)	14 (44)	0.453
**Body mass index**, kg/m^2	28 (25–31)	28 (25–31)	28 (24–33)	0.681
Body mass index ≥ 30 kg/m^2, *n*(%)	91 (32)	78 (31)	13 (41)	0.283
**FEV_1**, % predicted -mean (SD)	84 (22)	85 (22)	77 (20)	0.031
COPD, *n*(%)	171 (60)	151 (54)	20 (63)	0.779
**DL_{CO}**, % predicted -mean (SD)	83 (23)	84 (22)	78 (25)	0.082
**Epworth sleepiness scale**	6 (4–8)	6 (4–8)	6 (3–9)	0.980
**Apnoea–Hypopnoea index**, events/h	11 (4–22)	10 (4–21)	13 (4–27)	0.319
OSA, *n*(%)	167 (59)	146 (58)	21 (66)	0.405
**Oxygen desaturation index,** events/h	10 (4–21)	10 (4–21)	13 (6–26)	0.127
**Resting O_2 saturation**, %	93 (92–94)	94 (92–95)	93 (92–94)	0.026
**Median O_2 saturation**, %	92 (90–94)	92 (91–94	90 (89–93)	<0.001
**T90, %**	6 (1–30)	5 (1–25)	27 (2–70)	0.009
T90 ≥ 10%, *n*(%)	119 (42)	100 (40)	19 (59)	0.033
T90 ≥ 30%, *n*(%)	71 (25)	55 (22)	16 (50)	0.001
**% of lung with emphysema**	3 (1–7)	3 (1–7)	3 (1–7)	0.995
**Pulmonary artery diameter**, mm	26 (24–28)	25 (23–27)	30 (28–34)	<0.001
**Aorta artery diameter**, mm	34 (31–37)	34 (32–38)	30 (29–33)	<0.001
**PA:A ratio**	0.76 (068–0.84)	0.75 (0.67–0.81)	0.98 (0.93–1.01)	<0.001

Definition of abbreviations: Post-BD FEV_1 = post-bronchodilator forced expiratory volume in the first second; COPD = chronic obstructive pulmonary disease; DLco = diffusing capacity of the lung for carbon monoxide; OSA = obstructive sleep apnoea as defined by an apnoea–hypopnoea index ≥ 15 events·h $^{-1}$ (events/hour); T90 = percentage of sleep recording time with oxygen saturation <90%; PA:Ao ratio = ratio of the diameter of the pulmonary artery to the diameter of the ascending aorta. Data presented as median (25th–75th percentiles), mean (SD), or number (%). * *p* value differences were assessed using t tests or Mann–Whitney test for continuous variables and Pearson's Chi tests for categorical variables.

Table S4. Association of patient profile of severity of COPD and OSA and pulmonary artery enlargement as assessed by pulmonary artery diameter or PA:Ao ratio.

**Condition**	**Prevalence of Pulmonary Artery Enlargement (PAE)**		
	**Number (%)**	**PAE as defined by sex specific pulmonary artery diameter Number (%)**	**PAE as defined by the PA:Ao ratio Number (%)**
**COPD (*n*=171)**			
GOLD 1	77 (45)	9 (12)	7 (9)
GOLD 2	75 (44)	26 (35)	11 (15)
GOLD 3–4	19 (11)	12 (63) *	2 (11)
**OSA (*n*=167)**			
Mild (AHI, 5–15)	57 (34)	14 (25)	6 (11)
Moderate (AHI, 15–30)	65 (39)	11 (17)	8 (12)
Severe (AHI > 30)	45 (27)	18 (42)	7 (16)

Definition of abbreviations: COPD = Chronic Obstructive Lung Disease; OSA = obstructive sleep apnoea as defined by an apnoea–hypopnoea index (AHI) ≥ 5 events·h $^{-1}$ (events/hour); PA:Ao ratio = ratio of the diameter of the pulmonary artery to the diameter of the ascending aorta.

Table S5. Univariate associations with pulmonary artery enlargement as defined by the diameter of main pulmonary artery.

	OR	95% CI	*p*-Value
**Male sex**	1.37	0.78–2.39	0.267
**Age**, per 1-year increase	1.02	0.98–1.06	0.272
**Smoking history**, per 10 packs/year	1.01	1.00–1.03	0.021
**Current smoker**	0.95	0.57–1.59	0.849
**Body mass index**, per increase of 1 kg/m^2	1.08	1.03–1.14	0.001
**Obesity** (body mass index ≥ 30 kg/m^2)	2.39	1.38–4.13	0.002
**COPD**	1.15	0.67–1.98	0.613
**OSA**	0.92	0.54–1.57	0.763
**FEV_1 % predicted,** per 1 % decrease	1.03	1.02–1.04	<0.001
**DL_{CO}** % predicted, per 1 % decrease	1.01	0.99–1.02	0.309
**O_2 saturation at rest,** per 1% decrease	1.21	1.06–1.35	0.007
**Epworth sleepiness scale,** per 1-point increase	1.07	0.99–1.15	0.083
**Apnoea–Hypopnoea index**, per 1 event/h increase	1.01	0.99–1.03	0.077
**Oxygen desaturation index,** 1 event/h increase	1.02	1.01–1.04	0.045
**Median nocturnal SpO_2**, per 1% decrease	1.24	1.15–1.32	<0.001
**T90,** per 1 % increase	1.02	1.01–1.03	<0.001
**T90 ≥ 10 %**, yes vs. no	2.62	1.53–4.51	<0.001
**T90 ≥ 30 %**, yes vs. no	3.98	2.23–7.11	<0.001
**Lung volume with emphysema,** per 1% increase	1.01	0.97–1.04	0.959

Definition of abbreviations: OR = odds ratio; CI = confidence interval; FEV_1 = post-bronchodilator forced expiratory volume in the first second; COPD = chronic obstructive pulmonary disease; OSA = obstructive sleep apnoea as defined by an apnoea–hypopnoea index ≥ 15 events·h^{-1} (events/hour); DLco = diffusing capacity of the lung for carbon monoxide; T90 = percentage of sleep recording time with oxygen saturation <90%.
